# CD4+ Regulatory and Effector/Memory T Cell Subsets Profile Motor Dysfunction in Parkinson’s Disease

**DOI:** 10.1007/s11481-012-9402-z

**Published:** 2012-10-11

**Authors:** Jessica A. Hutter Saunders, Katherine A. Estes, Lisa M. Kosloski, Heather E. Allen, Kathryn M. Dempsey, Diego R. Torres-Russotto, Jane L. Meza, Pamela M. Santamaria, John M. Bertoni, Daniel L. Murman, Hesham H. Ali, David G. Standaert, R. Lee Mosley, Howard E. Gendelman

**Affiliations:** 1 Department of Pharmacology and Experimental Neuroscience, Center for Neurodegenerative Disorders, University of Nebraska Medical Center, Omaha, NE 68198-5880 USA; 2 Department of Neurology, University of Alabama at Birmingham, Birmingham, AL USA; 3 College of Information Science and Technology, University of Nebraska at Omaha, Omaha, NE USA; 4 Department of Neurological Sciences, University of Nebraska Medical Center, Omaha, NE USA; 5 Department of Biostatistics, University of Nebraska Medical Center, Omaha, NE USA; 6 Neurology Consultants of Nebraska, Omaha, NE USA; 7 Department of Pharmacology and Experimental Neuroscience Movement Disorders Program, University of Nebraska Medical Center, Omaha, NE 68198-5880 USA

**Keywords:** Parkinson’s disease, Motor function, Immune activation, T cells, CD4, Treg, Teff

## Abstract

Animal models and clinical studies have linked the innate and adaptive immune system to the pathology of Parkinson’s disease (PD). Despite such progress, the specific immune responses that influence disease progression have eluded investigators. Herein, we assessed relationships between T cell phenotype and function with PD progression. Peripheral blood lymphocytes from two separate cohorts, a discovery cohort and a validation cohort, totaling 113 PD patients and 96 age- and environment-matched caregivers were examined by flow cytometric analysis and T cell proliferation assays. Increased effector/memory T cells (Tem), defined as CD45RO+ and FAS+ CD4+ T cells and decreased CD31+ and α4β7+ CD4+ T cells were associated with progressive Unified Parkinson’s Disease Rating Scale III scores. However, no associations were seen between immune biomarkers and increased age or disease duration. Impaired abilities of regulatory T cells (Treg) from PD patients to suppress effector T cell function was observed. These data support the concept that chronic immune stimulation, notably Tem activation and Treg dysfunction is linked to PD pathobiology and disease severity, but not disease duration. The association of T cell phenotypes with motor symptoms provides fresh avenues for novel biomarkers and therapeutic designs.

**Electronic supplementary material:**

The online version of this article (doi:10.1007/s11481-012-9402-z) contains supplementary material, which is available to authorized users.

## Introduction

Parkinson’s disease (PD) is the most common neurodegenerative motor disorder (Hirtz et al. 2007). Loss of dopaminergic neurons and dopamine characterize the progressive loss of motor function and disease severity. Mounting experimental and clinical evidence has linked neuroinflammation to the pathobiology of PD (Mosley et al. 2012) whereby activated microglia and astrocytes comprise integral components of PD pathology (Pouplard and Emile 1984; Barker and Cahn 1988; McGeer et al. 1988; McGeer and McGeer 2008). Moreover, CD4+ and CD8+ T cells are found in close proximity to dopaminergic neurons in both PD brains (Brochard et al. 2009) and in 1-methyl-4-phenyl-1,2,3,6-tetrahydropyridine (MPTP)-treated mice (Kurkowska-Jastrzebska et al. 1999; Benner et al. 2008). In peripheral blood, decreased numbers of total lymphocytes are linked to decreased CD4+ T cell counts and percentages (Hoffman et al. 1978; Bas et al. 2001; Hisanaga et al. 2001; Baba et al. 2005; Calopa et al. 2010), which parallel reduced naïve and increased memory CD4+ T cells (Fiszer et al. 1994; Bas et al. 2001; Calopa et al. 2010), increased CD4^bright^CD8^dull^ T cells expressing CD45RO and FAS (Hisanaga et al. 2001), and increased CD4+ CD25+ T cells (Bas et al. 2001; Baba et al. 2005; Rosenkranz et al. 2007; Calopa et al. 2010). Although the cause and effects of such changes in T cell pools have not been precisely delineated, it is possible that T cells influence neurodegeneration. In support of this idea, modified forms of alpha synuclein (α-syn) are present in the periphery (Beach et al. 2010) and may act as neoantigens to break immune tolerance, and as such induce effector T cell (Teff) responses and subsequent neurotoxic reactions. Indeed, studies in our own laboratory demonstrated that induction of Teff speeds nigrostriatal degeneration following nitrated α-syn immunization (Benner et al. 2008). More recent works show that such effects are mediated through Th17 cells. These cells serve to exacerbate neurodegeneration while regulatory T cells (Treg) elicit neuroprotective responses as demonstrated in MPTP-intoxicated mice (Reynolds et al. 2010). In addition, Treg function is diminished in α-syn-immunized mice from which neurotoxic Th17 cells are isolated. Furthermore, decreased numbers of Treg are associated with increased rates of progression of amyotrophic lateral sclerosis (Beers et al. 2011; Rentzos et al. 2012) another neurodegenerative movement disorder.

Based on these findings, we hypothesized that alterations in the frequency, phenotypes and function of CD4+ T cells and CD4+ T cell subsets exist in PD, and that altered immune status co-exists with disease severity. Herein, we describe alterations in the peripheral CD4+ T cell, Treg, and Teff populations of PD patients compared with caregiver control subjects as well as with clinically-scored disease severity. Phenotypic markers for effector/memory T (Tem) cells were associated with clinical outcomes of disease severity, but not disease duration, and in vitro, Treg function was decreased. These data provide evidence of regulatory dysfunction with chronic activation of the adaptive immune system in PD which may have profound influence on ongoing inflammatory-induced neuropathology and disease progression associated with PD.

## Materials and methods

### Subjects and sample collection

Blood samples were obtained aseptically by venipuncture from PD patients *(n* = 113) and age- and environment-matched controls *(n* = 96), in two cohorts, a discovery cohort (Cohort A) and a validation cohort (Cohort B). The samples were assessed by flow cytometric analysis of peripheral blood mononuclear cells (PBMC) and used as sources for isolation of CD4+ T cell subsets. Participants were recruited through the University of Alabama at Birmingham (UAB) Movement Disorders Clinic, Neurological Consultants of Nebraska (NCNE), and the Department of Neurological Sciences at the University of Nebraska Medical Center (UNMC). Patients and controls provided written informed consent using IRB-approved forms. PD was diagnosed using UK Brain Bank clinical criteria. Patients and controls with a history of an autoimmune or inflammatory disorder and those receiving chronic immunosuppressive therapy were excluded. Controls were identified from among spouses and caregivers and are hereafter referred to as “caregivers”. A brief screening was conducted to exclude caregivers with symptoms likely to represent PD. Data on patients were collected using standard PD-DOC data forms: demographics, primary diagnosis, PD features, diagnostic features, family history, environmental risk, UPDRS-III, and Hoehn and Yahr (HY) stage. At UAB, 50 ml of whole blood were collected in acid citrate dextrose (ACD)-coated tubes (BD Vacutainer), coded and shipped with an ice pack overnight to the University of Nebraska Medical Center and processed within 24 h of collection. At NCNE and UNMC, 70 ml of whole blood were collected in heparin-coated tubes (BD Vacutainer), coded, and stored at room temperature until possessing, which occurred within 2 h of collection. Complete blood cell count with differential analysis was conducted on blood samples collected in EDTA-coated tubes (BD Vacutainer).

### Preparation of peripheral blood mononuclear cells and T cells

PBMC were collected by density gradient centrifugation using lymphocyte separation medium per manufacturer’s instructions (MP Biomedicals) and either used in proliferation assays, or frozen in fetal calf serum with 10 % dimethyl sulfoxide (DMSO) and stored in liquid nitrogen. Additionally, peripheral blood lymphocytes (PBL) were collected by elutriation of healthy donors, and enriched for naïve T cells using CD4+ T Cell Enrichment Columns (R&D Systems) following the manufacturer’s instructions with modifications including the addition of antibodies against CD25, CD8, and CD16 (BD Biosciences). Naïve T cells, greater than 94 % pure, were frozen in fetal calf serum with 10 % DMSO and stored in liquid nitrogen until use as responder T cells (Tresp) in proliferation assays. For proliferation assays, Tresp were thawed and labeled with carboxyfluorescein succinimidyl ester (CFSE) following the manufacturer’s instructions (CFSE, Molecular Probes).

### Cell sorting and flow cytometric analysis of phenotype and proliferative status

We performed multicolor flow cytometric analysis using a FACSCalibur flow cytometer (Becton Dickinson) with fluorochrome-conjugated monoclonal antibodies against the following antigens: CD4 (FITC or Alexa Fluor [AF]-700), CD25 (PE), CD127 (PerCP-Cy 5.5), FoxP3/Scurfin (AF-647), CD95/FAS/Apo1 (APC), CD31/PECAM-1 (AF647), CD39/ENTPD1 (APC), CD49d/Integrin α4 (APC or PE-Cy 7), CD103 (AF-647), CD45RO (APC), CD45RA (AF-700), Integrin β7 (APC), CD29/integrin β1 (AF700) (BD Biosciences, San Jose, CA, USA). Isotype-matched mouse or rat monoclonal antibodies were used as negative controls. Data analysis was conducted using BD FACSDiva Software version 6.1.3 (BD Biosciences, San Jose, CA, USA). In separate experiments, PBMC that were freshly-isolated from whole blood of PD patients and caregivers, were enriched for CD4+ T cells by negative selection with magnetic beads using AutoMACS (Miltenyi Biotec) per manufacturer’s instructions. Unstained lymphocytes were used as a negative control, and anti-mouse Ig, κ/Negative Control Compensation Particles (BD Biosciences) were used to optimize fluorescence compensation settings for fluorescence activated cell sorting (FACS) of CD4+ enriched T cells using a FACSAria II (Becton Dickinson). Naïve T cells were identified as CD4+CD25-CD127+, Treg were identified as CD4+CD25+CD127- and Teff were identified as CD4+CD25+CD127+, as expression of the alpha-chain of the IL-7 receptor, CD127, is inversely correlated with expression of FoxP3 and CD4+CD25+CD127-/low Treg are hypo-proliferative and suppressive (Liu et al. 2006; Seddiki et al. 2006). Cells isolated by FACS were plated with CFSE-labeled Tresp in RPMI 1640 medium supplemented with 10 % heat inactivated human AB serum (Atlanta Biologicals Inc.), 2 mM  L-glutamine, 55 uM 2-ME, 100 U/ml penicillin, and 100 μg/ml streptomycin, with 25 mM HEPES, 1 mM sodium pyruvate, 1X non-essential amino acids. T cells were activated by engagement of CD3 and CD28 with Dynabeads (Invitrogen), and proliferation was analyzed on day 3.5 by multicolor flow cytometric analysis.

### Gene expression analysis

PBMC were thawed and enriched for CD4+ T cells by negative selection with magnetic beads using AutoMACS (Miltenyi Biotec) per manufacturer’s instructions. CD4+ T cells were then cultured for 20 h in RPMI 1640 medium supplemented with 10 % heat inactivated human AB serum, 2 mM L-glutamine, 55 μM 2-ME, 100 U/ml penicillin, and 100 μg/ml streptomycin, with 25 mM HEPES, 1 mM sodium pyruvate, 1X non-essential amino acids with anti-CD3/CD28 Dynabeads (Invitrogen). mRNA was isolated using a RNeasy Mini Kit with on-column DNase digestion on a QIAcube according to the manufacturer’s protocol (QIAGEN). RNA was stored at −80° C in RNase-free water. PCR reactions were conducted using the RT^2^ Profiler PCR Array Human Th1-Th2-Th3 with RT^2^ SYBR Green qPCR Master Mix on an Eppendorf realplex Mastercycler ep gradient S (SABiosciences a QIAGEN Company).

### Bioinformatics correlation network creation via node seeding

Cell surface markers that were differentially expressed by flow cytometric analysis in the PD group compared to caregivers were used to build networks for correlation analysis. Nodes in the networks represent cell surface markers. Edges represent the weighted correlation of each gene and an associated p-value. Correlations with *p*-value >0.0005 were not considered statistically significant and edges outside that threshold were discarded; all correlations met this threshold. Edge width and opacity refers to strength of correlation (positive or negative correlation).

### Statistical analysis

Fisher’s exact tests were used to examine the association of categorical participant characteristics with disease status. A two-sample *t*-test was used to examine the association of age with disease status. Normality of flow cytometric data was assessed using the Anderson-Darling test. Since many of the flow cytometric measures did not pass the test for normality, values were compared between caregivers and PD patients using a non-parametric Mann–Whitney test. To control for the false discovery rate at the α level, p values obtained from comparing flow cytometric data of caregivers and PD patients were adjusted using Benjamini–Hochberg adjustment for multiple comparisons. Pearson product–moment correlation coefficients were used to determine correlations between antigens measured by flow cytometric analysis and UPDRS-III score, and FAS and CD45RO. Linear regression was used to determine if age or UPDRS-III score predict variables measured by flow cytometry. Associations of flow cytometric data (CD45RO+RA−, FAS and α4β7) with UPDRS-III scores (binned into 3 groups), were assessed using Kruskal–Wallis non-parametric ANOVA with post hoc pairwise comparisons of PD patients verses controls conducted with Dunn’s multiple comparison test. Regression analysis was used to adjust for gender; a general linear model on rank-transformed CD31 values was used to evaluate CD31 and UPDRS-III followed by a Bonferroni adjustment for multiple comparisons. Statistical analyses were conducted using Prism (GraphPad Software, Inc) or IBM SPSS (IBM).

## Results

We collected data from two cohorts of PD patients and caregivers; a discovery cohort (Cohort A) and a validation cohort (Cohort B). Descriptive statistics of Cohorts A and B demonstrate that gender, and self-reported exposure to pesticides (Cohorts A and B), chemical solvents and heavy metals (Cohort B) were associated with PD (supplemental Table S1). More importantly, both cohorts were comprised of PD patients and caregivers that were similar between cohorts with respect to age, disease duration, motor function and disease severity; the latter two scored by practicing neurologists using part III of the United Parkinson’s Disease Rating Scale (UPDRS), the most commonly used assessment of disease severity (Leddy et al. 2011). In the pilot study, Cohort A, whole blood samples from PD patients (*n* = 41) and caregivers (*n* = 31) were evaluated by flow cytometric analysis in an un-blinded fashion to investigate phenotypic leukocyte antigens of interest. Those analyses suggested that peripheral immune T cell phenotypic changes resided in the CD4+ T cell, CD4+CD25+CD127- Treg and CD4+CD25+CD127+ Teff populations in PD, and that the percentage of effector/memory T cells (Tem) were increased (supplemental Table S2). Based on these preliminary results, a prospective blinded study was designed for a second, but larger, validation cohort, Cohort B, consisting of 72 PD patients and 65 caregivers (supplemental Table S1).

### Lymphocyte and CD4+ T cell frequencies in PD

**Fig. 1:**
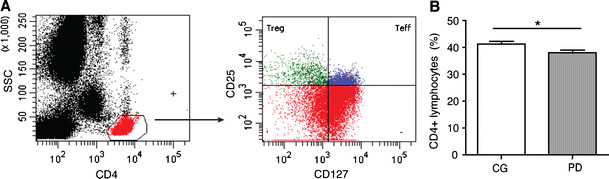
Gating strategy for flow cytometric analysis of PBMC and CD4+ T cell and Teff frequency*.* **a** Representative flow cytometric scatter plots used for data collection. The CD4+ T cell population was identified by high expression of CD4 and low side scatter (left panel). Treg and Teff were identified within the CD4+ T cell population as CD25+CD127− and CD25+CD127+ (right panel). **b** The percentage of CD4+ lymphocytes for Cohort B. CD4+ data (**b**) are expressed as means ± SEM, and significant differences between CD4+ T cell means were determined by Mann–Whitney test for 63 caregivers and 71 PD patients where *p ≤ 0.05

To define changes in the numbers and phenotype of CD4+ T cells, Treg and Teff of PD patients in Cohort B, we conducted flow cytometric analyses of peripheral blood mononuclear cells (PBMC) from PD patients and caregivers. CD4+ T cell populations were identified by high expression of CD4 and low side scatter, and Treg and Teff were identified within the CD4+ T cell population as CD25+CD127- and CD25+CD127+, respectively (Liu et al. 2006; Seddiki et al. 2006) (Fig. 1a). To confirm identification of Treg, the intracellular transcription factor forkhead box P3 (FoxP3) was measured. As expected, the percentage of FoxP3+ Treg was consistently and significantly higher than the percentage of FoxP3+ Teff in both PD patients and caregivers, suggesting that CD4+CD25+CD127- T cells are Treg (Liu et al. 2006) (supplemental Fig. S1). CD4+ T cell frequency was decreased in PD patients compared to caregivers *(*Fig. 1b), while no significant differences were seen in the percentages of Treg and Teff amongst PD patients and caregivers (supplemental Fig. S1).

To determine whether the decreased percentage of CD4+ T cells in cohort B was due to reductions in the absolute number of CD4+ T cells or an increase in other lymphocyte populations, complete blood count (CBC) and differential counts of PD patients were compared to caregivers. Hemoglobin concentrations, total white blood cell, absolute lymphocyte and CD4+ T cell counts were assessed for Cohort B (Table 1). Compared to caregivers, PD patients presented diminished percentages of lymphocytes and decreased absolute lymphocyte counts with increased percentages of neutrophils. Using the CD4+ T cell percentages from flow cytometric analyses and absolute lymphocyte counts from differentials, we calculated absolute CD4+ T cell counts, which were significantly decreased in PD patients compared to caregivers.

**Table 1:** Complete blood counts and differential counts from PD patients compared to caregiver controls of Cohort B

	Caregivers				PD patients				*p* values
	n	Mean ± SEM	Median	Range	n	Mean ± SEM	Median	Range	Mann–Whitney
Hgb (g/dL)	47	13.7 ± 0.2	13.8	11.1–17.3	59	14.1 ± 0.2	14	11.6–17.3	0.08
WBCx 10^3^/μL	47	6.4 ± 0.3	6	3.6–11.4	59	6.2 ± 0.2	6	3.3–12.1	0.57
Neutrophil (%)	36	57.5 ± 1.8	60	27–77	43	63.6 ± 1.5	64	43–86	0.01
Lymphocyte (%)	36	29.7 ± 1.6	28.5	10–59	43	24.1 ± 1.2	23	7–38	0.01
Monocyte (%)	36	8.6 ± 0.4	8	5–14	43	8.4 ± 0.3	8	4–13	0.75
Eosinophil (%)	36	3.7 ± 0.3	3	0–9	43	3.4 ± 0.5	3	0–13	0.70
Basophil (%)	36	0.6 ± 0.1	1	0–1	43	0.4 ± 0.1	0	0–2	0.12
Absolute Lymphocyte count/μL	36	1829.3 ± 174	1537.5	737–6726	43	1434.6 ± 89.4	1340	570–3335	0.04
^a^Absolute CD4+ count/μL	36	801.3 ± 115.4	591.5	334.3–4424	43	541.3 ± 37.9	469.4	207–1231	0.008

^a^Absolute CD4+ T cell count was calculated using the percentage of CD4+ T cells determined by flow cytometric analysis with the absolute lymphocyte count determined by differential

### Increased effector/memory CD4+ T cells in PD

Phenotypic changes in the CD4+ T cell, Treg and Teff populations were assessed by flow cytometric analysis (Table 2). CD4+ T cells from PD patients demonstrated increased percentages of CD45RO+ events and FAS+ events than caregivers, while the percentages of CD45RA+ and CD31+ CD4+ T cell events were decreased in PD. The percentages of integrin α4β7+ cells were decreased significantly in PD patients, and integrin α4β1+ CD4+ T cells were elevated slightly, though not significantly *(p* = 0.08). Within the Teff and Treg populations, FAS+ Teff were increased and CD45RA+ Teff were significantly decreased in PD patients compared to caregivers, while there were no differences observed in the Treg population. Significant differences in phenotypic markers of CD4+ T cell subsets determined by Mann–Whitney comparison remained significant after Benjamini-Hochberg adjustment for multiple comparisons (Table 2). Although gender was associated with disease status (Table S1), the percentages of CD45RO+, FAS+ and integrin α4β7+ events in the CD4+ T cell population of the PD group remained significantly different from that of the caregiver group after adjusting for gender. However, the percentage of CD4+ T cells and the percentages of CD4+ T cells expressing CD45RA, CD31, and integrin α4β1 were not significantly different after adjusting for gender.

**Table 2:** Phenotypic analysis of CD4+ T cells and subsets from PD patients compared to caregivers in Cohort B

Population	Population Subset	Caregivers				PD patients				*p*-values	
		n	Mean ± SEM (%)	Median (%)	Range (%)	n	Mean ± SEM (%)	Median (%)	Range (%)	Mann–Whitney	Benjamini-Hochberg
CD4+ T cells	CD45RO+	62	64.4 ± 1.5	65.2	37.6–93.4	71	69.6 ± 1.5	69.7	22.4–91.4	0.009	0.028
	CD45RA+	62	27.5 ± 1.4	27.8	3.0–54.0	71	22.4 ± 1.5	21.4	2.1–72.8	0.004	0.028
	FAS+	65	60.0 ± 1.8	61.2	27.7–96.1	72	67.0 ± 1.7	67.6	22.4–93.3	0.006	0.028
	CD31+	65	28.8 ± 0.9	28.1	14.7–47.3	72	25.3 ± 0.9	25.6	10.6–41.2	0.014	0.031
	Integrin α4β7+	53	37.6 ± 1.5	36.6	20.8–60.0	58	31.8 ± 1.3	33.3	12.0–55.6	0.021	0.034
	Integrin α4β1+	41	52.5 ± 1.5	50.7	38.2–78.2	47	55.6 ± 1.6	54.5	34.4–82.0	0.08	0.10
	CD25+	64	13.2 ± 0.4	13.0	6.8–21.1	71	13.7 ± 0.4	13.3	7.1–20.8	0.48	0.54
	CD127+	64	63.7 ± 0.8	64.0	44.0–77.0	71	64.2 ± 0.8	64.7	44.9–79.6	0.66	0.66
CD4+CD25+ CD127+ Teff	CD45RO+	60	88.1 ± 0.8	89.9	69.4–97.6	70	89.2 ± 0.9	91.6	57.0–98.4	0.07	0.08
	CD45RA+	60	5.2 ± 0.6	3.7	0.2–20.4	70	4.6 ± 0.7	2.8	0.2–33.9	0.05	0.06
	FAS+	63	89.9 ± 1.3	92.2	35.4–99.3	71	92.7 ± 0.8	95.0	60.9–98.8	0.04	0.06

CD45RO+CD4+ T cells increase with age (Douek et al. 1998). However, the mean age of PD patients compared to caregivers were not significantly different (Table S1), suggesting that the increase in the percentages of CD45RO+CD4+ T cells in PD patients compared to caregivers was not age-associated. Previous studies have demonstrated that a high percentage of CD45RO+ memory CD4+ T cells are FAS+ (Miyawaki et al. 1992), and as expected, regardless of diagnosis, the percentages of CD45RO+CD4+ T cells and FAS+CD4+ T cells were found to be strongly correlated (supplemental Fig. S2A). In addition, we found a moderate negative correlation between percentages of CD45RO+CD4+ T cells and CD31+ CD4+ T cells among all Cohort B participants (supplemental Fig. S2B), and found a weak negative correlation between the percentages of CD31+ and FAS+CD4+ T cells (supplemental Fig. S2C).

### Association of disease severity with T cell phenotypes

We assessed the relationship of phenotypic alterations in CD4+ T cells, Treg and Teff with age, clinical measures of disease severity and disease duration. Linear regression demonstrated that age was not predictive of the variation in the percentage of CD4+ T cells that were CD45RO+ in PD patients (*r* ^2^ = 0.044, *p* = 0.13) or caregivers (*r* ^2^ = 0.003, *p* > 0.05) (data not shown). We found a moderate positive correlation between UPDRS-III score and the percentages of CD45RO+CD4+ T cells (Fig. S3A), and a weak positive correlation between UPDRS-III score and the percentage of FAS+CD4+ T cells (Fig. S3B). The percentages of CD31+ CD4+ T cells demonstrated a strong negative correlation with UPDRS-III (Fig. S3C) and integrin α4β7+ CD4+ T cells showed a moderate negative correlation with UPDRS-III (Fig. S3D). CD45RO expression by Teff was weakly correlated with UPDRS-III (Pearson *r* = 0.24, data not shown), while CD31 expression on Teff showed a strong negative correlation with UPDRS-III score (Fig. S3E), and α4β7+ Teff were show a moderate inverse correlation with UPDRS-III score (Fig. S3F). No correlations of disease duration as measured by years since diagnosis could be established with the percentages of CD45RO+, FAS+, CD31+, or integrin α4β7+ CD4+ T cells (data not shown).

**Fig. 2:**
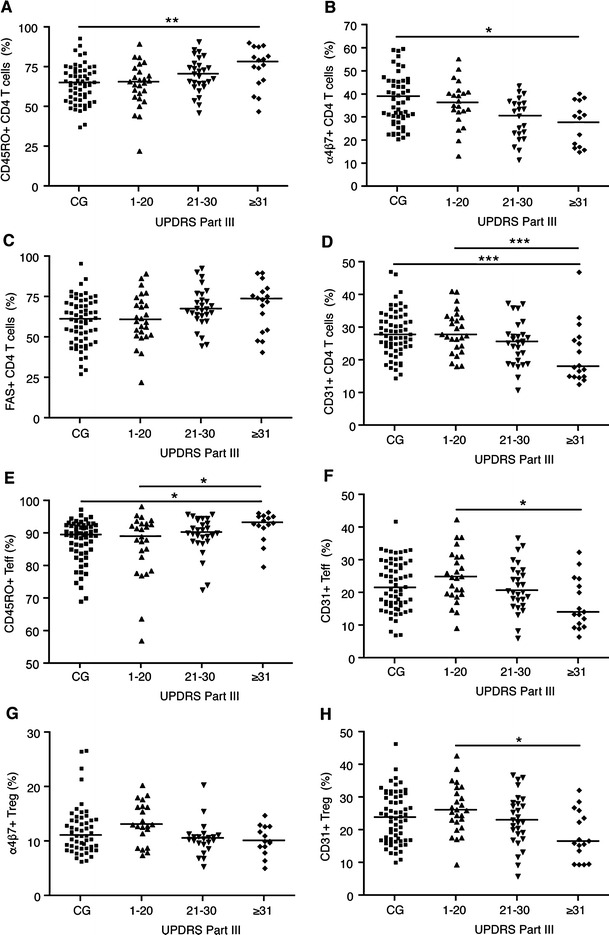
CD4+ T cell and Teff phenotypes are associated with UPDRS-III score. Flow cytometric data of caregivers and PD patients from Cohort B were binned into 4 groups based on UPDRS-III scores: caregivers (CG, *n* = 61), 1–20 *(n* = 25), 21–30 *(n* = 28), and ≥31 *(n* = 16). The percentages of CD4+ T cells expressing CD45RO (**a**), α4β7 (**b**), FAS (**c**), and CD31 (**d**) in each group were associated with UPDRS-III group (*p* < 0.05). Percentages of CD45RO+ (**e**) and CD31+ (**f**) Teff and the percentages of α4β7+ (**g**) and CD31+ (**h**) Treg in each group were associated with UPDRS-III group (*p* < 0.05). Data are the percent-positive of T cells with medians (*horizontal lines*). Significant differences among groups were determined by Kruskal-Wallis nonparametric ANOVA (CD45RO, α4β7 and FAS) or by general linear model (CD31), and pair-wise comparisons were determined by either Dunn’s or Bonferroni adjustments for multiple comparisons (CD31) where * *p* ≤ 0.05, **p ≤ 0.01, and ***p ≤ 0.001

To further investigate the relationship between disease severity and CD4+ T cells, we next assessed flow cytometric data of caregivers and PD patients segregated into 3 groups based on UPDRS-III scores of: 1–20 *(n* = 25), 21–30 *(n* = 28), and ≥31 *(n* = 16). Nonparametric ANOVA indicated differences among groups with respect to the percentages of CD45RO+, α4β7+, and FAS+CD4+ T cells (Fig. 2a-c), and Dunn’s adjustment for multiple comparisons demonstrated that the significant differences occurred between caregivers and PD patients with a UPDRS-III score ≥31 with respect to the percentages of CD45RO+ and α4β7+ CD4+ T cells. After adjusting for gender, there was a significant association between CD31 and UPDRS-III score, and Bonferroni adjustment for multiple comparisons revealed a significant difference between controls and PD patients with UPDRS-III score ≥31 and PD patients with a UPDRS-III score of 1–20, compared to those with a UPDRS-III score ≥31 (Fig. 2d). In the Teff population, CD45RO+ was associated with UPDRS-III, with significant differences occurring between PD patients with a UPDRS-III score ≥31 and caregivers and those with a score between 1 and 20 (Fig. 2e). After adjusting for gender, percentages of CD31+ CD4+ T cells were associated with UPDRS-III, and Bonferroni adjustment for multiple comparisons demonstrated that PD patients with UPDRS-III scores of 1–20 were significantly different from those with a score ≥31 (Fig. 2f). In the Treg population, the percentages of CD45RO+ and FAS+ cells were not associated with UPDRS-III scores, but α4β7+ Treg percentages were associated with UPDRS-III (Fig. 2g). After adjusting for gender, percentages of CD31+ Treg were associated with UPDRS-III scores, wherein Bonferroni adjustment for multiple comparisons demonstrated that the percentages of CD31+ Treg in PD patients with a UPDRS-III score ≥31 were significantly less than those from patients with scores of 1–20 (Fig. 2h).

### Treg suppressive function, but not CD4+ T cell proliferative capacity is affected in PD

**Fig. 3:**
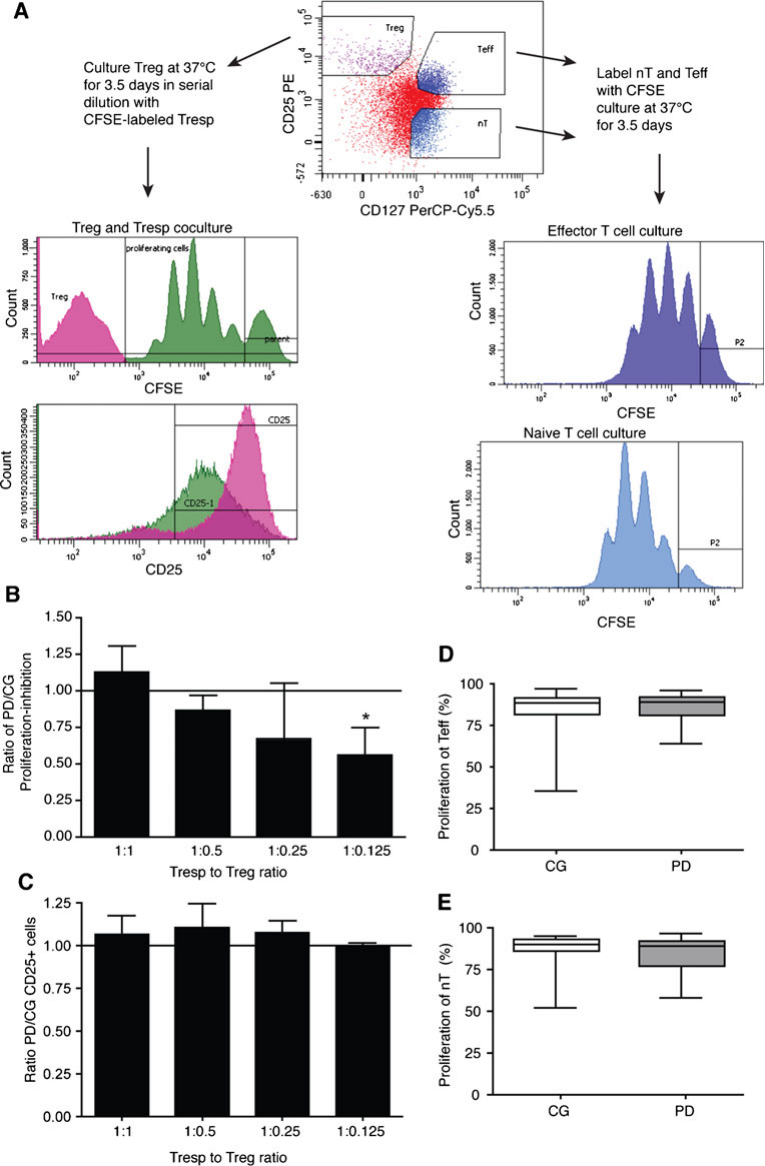
Treg from PD patients are dysfunctional while naïve T cells (nT) and Teff show no alterations in proliferative capacity. **a**Treg, nT and Teff were identified within the CD4+ T cell population as CD25hiCD127-, CD25-CD127+, and CD25+CD127+, respectively (*top, center dot plot*). CFSE-labeled CD4+CD25− allogeneic Tresp were co-cultured with serially-diluted Treg. By flow cytometric analysis, CFSE was measured to determine the percentage of Tresp that proliferated, and the percentage of CD25+ Tresp was measured as an indication of activation (histograms, left panels). The proliferative capacity of isolated CFSE-labeled Teff and nT was measured by flow cytometric analysis of CFSE (histograms, right panels). **b** Inhibition of proliferation of Tresp by Treg at ratios of 1:1 *(n* = 28), 1:0.5 *(n* = 26), 1:0.25 *(n* = 28) and 1:0.125 *(n* = 25) of Tresp to Treg, demonstrating that Treg from PD patients had decreased inhibitory capacity at the 1:0.125 dilution (*p* = 0.006). **c** CD25 expression was not altered at any dilution. **d** The percentage of proliferating Teff did not differ (*p* < 0.05) for PD patients *(n* = 34) compared to caregivers *(n* = 32). **e** The percentage of proliferating nT did not differ (*p* < 0.05) for PD patients *(n* = 16) compared to caregivers *(n* = 15). Data are expressed as the percentage of proliferating cells out of all CFSE+ events. Significant differences among groups were determined by Kruskal-Wallis nonparametric ANOVA, and pair-wise comparisons determined by Dunn’s multiple comparison’s post-hoc analysis (**b**, **c**) or by Mann–Whitney test (**d**, **e**)

We next conducted in vitro functional assays to determine whether Treg isolated from PD patients had reduced ability to suppress the proliferation of CD4+CD25- responder T cells (Tresp) from healthy allogeneic donors. CD4+CD25hiCD127- Treg, CD4+CD25-CD127hi naïve T cells and CD4+CD25+CD127hi Teff were isolated by fluorescence-activated cell sorting from PBMC of PD patients and caregivers (Fig. 3a). Treg were serially-diluted and co-cultured with anti-CD3/anti-CD28-coated beads and a constant number of CSFE-labeled Tresp. As indicators of T cell activation and proliferation, we measured CD25 for the former and the loss of CSFE for the latter by flow cytometric analysis (Fig. 3a, left histograms). We first tested whether Treg from PD patients compared to caregivers would equally suppress proliferation of Tresp at all dilutions (PD/CG, Treg-mediated suppression = 1). We found that PD Treg showed decreased ability to suppress Tresp proliferation at the greatest dilution (1:0.125) (Fig. 3b). This raises the possibility that microenvironments in which Treg are greatly outnumbered, differentially inhibit Treg function in PD compared to caregivers. CD25 expression on T cells increases upon activation (Depper et al. 1984), and thus should be inhibited in the presence of Treg. Indeed, CD25 expression was correlated with Treg dilution for PD patients (*r* ^2^ = 0.48, *p* < 0.001) and caregivers (*r* ^2^ = 0.67, *p* < 0.001). However, Treg from PD patients did not suppress expression of CD25 differentially than caregivers (Fig. 3c, PD/CG, Treg-mediated suppression = 1). To determine if the increase in memory T cell phenotype in PD is due to hyperproliferative naïve T cells (nT) or effector T cells (Teff), we isolated and measured the proliferative response of Teff and nT after CD3/CD28 stimulation (Fig. 3a, right histograms). No significant differences in proliferative capacity of Teff (Fig. 3d) or nT (Fig. 3e) were found between PD patients and caregivers suggesting that aberrant proliferation of nT or Teff does not contribute to the phenotypic skewing towards Tem.

### CD27 mRNA is decreased, while IL-9 and IL-6 are increased in CD4+ T cells from PD patients

To elucidate potential causes of phenotypic changes in the CD4+ T cell population, we conducted quantitative reverse transcription polymerase chain reaction (qRT-PCR) for gene expression associated with helper T cell phenotypes in CD3/CD28-activated CD4+ T cells from PD patients *(n* = 7) and caregivers *(n* = 9). mRNA levels of the anti-apoptotic cytokine, IL-9, were increased by 3.1-fold. IL-6 mRNA levels were significantly increased by 2.3-fold, while CD27 mRNA expression was diminished by 1.6-fold (data not shown). Memory T cells have been shown to increase IL-9 production after anti-CD3/CD28 stimulation in vitro (Soler et al. 2006). IL-6 is associated with chronic inflammatory responses (Gabay 2006), and CD27 expression is reduced on mature lymphocytes (Hintzen et al. 1993). Thus, these data further support the results demonstrating chronic inflammatory responses in PD and skewing of CD4+ T cells towards the Tem phenotype.

### Bioinformatics networks propose a PD immunophenotype

**Fig. 4:**
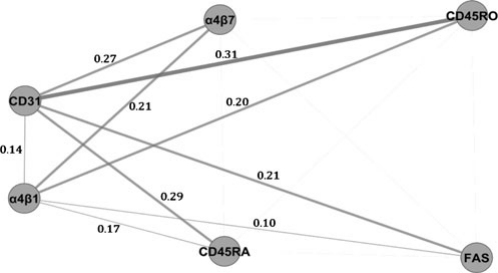
Correlation difference networks of caregivers and PD patients. Differences network interactions are demonstrated by the correlation difference network, where edge width and opacity reflects the correlation difference score (*n* = 37 CG, 46 PD). Correlations with *p*-value >0.0005 were not considered statistically significant, and edges outside that threshold were discarded

Flow cytometric analyses above (Table 2, Fig. 2 and Fig. S3) suggest that phenotypic markers of Tem are linked to PD. To investigate associations among phenotypic markers of Tem, we utilized bioinformatics to conduct pair wise computations of Pearson correlations for each possible combination of phenotypic markers within each dataset (PD patient and caregiver). The absolute differences in the correlation scores of the edges of the PD and caregiver networks were calculated and are presented as a Correlation Difference Network (Fig. 4). The network structures for both the PD and caregiver groups were conserved fairly strongly. While the edges emanating from CD31 consisted of the strongest differences in correlation, the association of CD31 with gender most likely contributes to this difference. The weaker correlation coefficient of α4β1 with α4β7 in PD compared to that of caregivers suggest that the increased variation in the CD4 T cell pool of PD patients is due to the increased percentage of integrin α4β7+ CD4+ T cells. Similarly, the difference in the correlation coefficients of α4β1 with CD45RO in PD patients compared to caregivers is likely due to the increased percentage of CD45RO+ CD4+ T cells in PD. Thus, these data further support the accumulation of Tem in PD patients.

## Discussion

This report demonstrates associations between PD, environmental exposures, gender, effector memory T cells (Tem) and Treg function. While previous studies have found alterations in PD lymphocyte and CD4+ T cell populations, we now confirm and extend these results by demonstrating that changes in CD4+ T cell, Treg and Teff phenotypes are associated with motor function scores determined by UPDRS-III; the most commonly used assessment of disease severity (Leddy et al. 2011). Moreover, PD patients with UPDRS-III scores of 30 or higher had increased CD45RO+ and FAS+CD4+ T cells and decreased α4β7+ and CD31+ CD4+ T cells; indicative of increased effector/memory T cells. Despite best medical management provided to PD patients, UPDRS scores increase over time and parallel disease progression (Holloway 2009). Thus, the UPDRS-III “on medication” score is a reasonable proxy for disease severity. This report is the first, to our knowledge, that has associated PD motor severity with T cell phenotypes. Specifically, the predominance of Tem in more severe stages of disease supports a role of chronic immune activation in disease progression.

A principal question that arises from these studies is how can this occur? PD is a disease of the nervous system and engagement of peripheral T cell responses seems ill connected. However, mounting evidence implicates both the innate and adaptive immune systems in the pathobiology of PD. Aberrant species of α-syn in Lewy bodies are linked to microglial activation, oxidative stress, neuroinflammation, and loss of dopaminergic neurons in affected brain regions (Czlonkowska et al. 1996; Zhang et al. 2005; Reynolds et al. 2008). The same protein species are also found in the periphery (Beach et al. 2010). Nitrated-α-syn, but not unmodified α-syn is found in cervical lymph nodes of MPTP-treated mice (Benner et al. 2008), and modified forms of α-syn are in gut tissue of PD patients (Lebouvier et al. 2010); Forsyth et al. 2011). The presence of modified forms of α-syn in lymph nodes and gut tissues present a means for neoantigen exposures and activation of the adaptive immune system. To test this hypothesis, we previously investigated whether N-α-syn-specific T cells adoptively transferred to MPTP-intoxicated recipient mice could play a role in nigrostriatal degeneration. We observed that vasoactive intestinal peptide (VIP)-induced natural regulatory T cells (Treg) were neuroprotective, while N-α-syn-specific helper T cells (Th1 and Th17) exacerbated MPTP-induced neuronal degeneration (Reynolds et al. 2010). Therefore, we further hypothesized that alterations in the frequency, phenotypes or function of CD4+ T cells, Treg and Teff are operative in PD patients and associated with disease severity or duration. This study demonstrates that T cell phenotypes, specifically those of the effector/memory lineage, are associated with clinical outcomes of disease severity.

It was previously suggested that the relative lymphopenia in PD could be caused by FAS-mediated apoptosis related to CD25 (Bas et al. 2001). Indeed, we observed increased lymphocyte FAS expression in PD patients as previously reported (Calopa et al. 2010). However, increases in apoptosis may not be due to FAS alone (Calopa et al. 2010). In addition to an increase in FAS, we observed diminished CD31 expression, which was associated with disease severity. CD31, or platelet endothelial cell adhesion molecule-1 (PECAM-1), is expressed on most naïve CD4+ T cells, but is decreased on naïve T cells undergoing homeostatic proliferation (Azevedo et al. 2009) and on effector memory T cells (Ashman and Aylett 1991; Demeure et al. 1996), particularly those activated by the T cell receptor (Kohler and Thiel 2009). Using qRT PCR, we found no detectable diminution of CD31 mRNA transcripts in PD patients; a finding that is not unexpected since CD31 is not regulated at the transcript level (Fornasa et al. 2010). Under normal conditions, CD31-signaling controls the amplitude of clonal expansion and is required for establishment of regulatory functions and T cell tolerance (Lebouvier et al. 2010) by negatively regulating TCR-mediated signal transduction (Newton-Nash and Newman 1999; Kohler and Thiel 2009). Recent studies using CD31 deficient mice support the role of CD31 in controlled T cell activation and survival, and demonstrate that loss of CD31 increases T cell susceptibility to apoptosis (Ross et al. 2011). Thus, the decrease in CD31 on PD CD4+ T cells, particularly in those with advanced motor dysfunction, may contribute to the decrease in CD4+ T cell counts by increasing apoptosis. In addition, while CD31^−/−^ mice are known to display normal numbers of CD25+FoxP3+ Treg, CD31^−/−^ Treg have impaired regulatory function at low Treg:Tresp ratios (Lebouvier et al. 2010). Here, we found that the percentages of CD31+ Treg in PD patients negatively correlated with disease severity, and Treg function was reduced at low Treg:Tresp ratios. The negative correlation and association of CD31 with disease severity and the memory phenotype suggest that decreased CD31 may be attributed to increased T cell activation (Kohler and Thiel 2009), yet it is possible that the loss of CD31 is also aberrant and contributes to decreased Treg function in PD as seen in CD31^−/−^ mice.

Our results support previous studies demonstrating an increase in CD45RO+CD4+ T cells in PD (Fiszer et al. 1994; Bas et al. 2001; Calopa et al. 2010). However, we observed additional markers of memory T cells that were differentially expressed in PD. The observed increase in CD45RO+ and decrease in CD45RA+CD4+ T cells paralleled the increase in FAS+CD4+ T cells and decrease in CD31+ CD4+ T cells, which are characteristics of activated or proliferating T cells (Oyaizu et al. 1994; Demeure et al. 1996). In this context, decreased expression of CD27 at the mRNA level in the CD4+ T cell population suggests that the Tem phenotype is increased in PD (Hintzen et al. 1993). Tem cells and central memory T cells (Tcm) are distinct subsets of memory T cells defined by function (Pepper and Jenkins 2011). The Tem pool contains Th1, Th2, and cytotoxic T cells that migrate to inflamed peripheral tissues and have immediate effector function, while Tcm home to secondary lymphoid tissues and have little effector function but are able to proliferate and differentiate into effector T cells (Th1, Th2, Th17) in response to antigenic stimulation (Sallusto et al. 2004). Both memory T cell subsets are highly responsive to antigenic stimulation but have reduced proliferative capacity and an increased propensity to undergo apoptosis (Sallusto et al. 1999, 2004). This lends further support to the observed relative lymphopenia and reduced CD4+ T cell numbers in PD found here and previously. The co-stimulatory molecule, CD27 functions to promote survival of activated and memory T cells and to generate the effector T cell pool (Hendriks et al. 2003). However, memory T cells acquire the CD45RA-CD27- Tem phenotype after chronic antigenic stimulation (De Jong et al. 1992). These data support a predominating Tem cell phenotype in PD patients, which is a significant finding as it strongly supports the hypothesis that PD has chronic inflammatory components at play in the periphery. This notion is further supported by the observation that chronic infection, and thus chronic antigenic stimulation, leads to decreased expression of CD31 and CD27 (Yonkers et al. 2011).

We also observed a significant decrease in α4β7+ CD4+ T cells and a slight increase in α4β1+ CD4+ T cells in PD patients compared to caregivers. Low expression of α4β7 and high expression of α4β1 are characteristic of brain-tropic T cells (Denucci et al. 2009), as the interaction between α4β7 and mucosal addressin cell adhesion molecule 1 (MAdCAM-1) allows for entry into the gut (Agace 2006), and α4β1 and vascular cell adhesion molecule 1 (VCAM-1) on endothelial cells allows for entry of T cells into the brain (Engelhardt and Ransohoff 2005). While the observed increase in α4β1+ CD4 T cells in the current study was not significant and was associated with gender, the significant decrease in α4β7+ CD4 T cells alone may nonetheless be indicative of brain tropic T cells. Alternatively, these data may suggest an increase in inflammatory responses in the gut. Increased expression of MAdCAM-1 on endothelial cells in inflamed gut tissue augments α4β7+ T cell homing and compartmentalization to the gut, leading to decreased frequency of in α4β7+ T cells in the peripheral blood (Di Sabatino et al. 2009). No studies have yet to investigate inflammation per se in gut tissue of PD patients. However, studies of PD gut tissue have demonstrated the presence of proinflammatory immune mediators (Lebouvier et al. 2010; Forsyth et al. 2011). Histopathological studies of PD gut mucosa demonstrate increased intestinal permeability, which correlated with *E. coli* bacteria, nitrotyrosine and α-syn staining (Forsyth et al. 2011). Furthermore, non-motor symptoms can precede PD diagnosis by several years or decades and persist as the disease progresses (Strang 1965). In the context of these prodromes and Braak staging of Parkinson’s disease neuropathology, our findings of chronic inflammation in the periphery strengthens the “dual hit” theory, which suggests that the etiology of PD may be infection by a pathogen that gains entry to the CNS through the periphery (e.g., nasal and gut tissues) (Hawkes et al. 2007).

In summary, we posit that a chronic-inducer of T cell stimulation in the periphery exists in PD and provides an association between the adaptive immune system activity and motor dysfunction. Notably, we observed that immunological markers of chronic T cell activation are associated with disease severity, but not age or duration of disease. As UPDRS-III scores (i.e., motor dysfunction) increase, the Tem cell phenotype, indicative of chronic activation, predominates. CD45RA expression decreases while CD45RO expression increases; cell surface expression of α4β7 and CD31 decline while FAS expression increases, and CD27 transcription levels decrease. The decrease in CD31 in combination with increased FAS, contributes to apoptosis and the subsequent relative lymphopenia. In addition, the decrease in CD31 on Treg in PD patients with more severe motor dysfunction, may contribute to impaired suppressive function at lower Treg:Tresp ratios. Altogether, these data combined with recent reports of increased intestinal permeability and the presence of modified α-syn, Lewy body and infectious inflammatory mediators in the PD gut tissue, lend support to the “dual hit” theory whereby peripheral engagement of antigens such as modified-self α-syn affect disease progression.

## Electronic supplementary material

Below is the link to the electronic supplementary material.

Fig. S1 The percentage of FoxP3+ Treg and Teff from PD patients and caregivers. Data are the percentages of FoxP3 positive Treg and Teff with medians (horizontal lines). Significant differences among groups were determined by Kruskal-Wallis nonparametric ANOVA, and pair-wise comparisons determined by Dunn’s multiple comparison’s post-hoc analysis where ***p ≤ 0.001. (JPEG 14 kb)

High resolution (TIFF 151 kb)

Fig. S2 The percentages of CD45RO+, FAS+ and CD31+ CD4+ T cells are correlative. **a** Scatter plot of the percentage of FAS+ CD4+ T cells against the percentage of CD45RO+ of CD4+ T cells for Cohort B (Pearson *r* = 0.87, *p* < 0.001). **b** Scatter plot of the percentage of CD31+ CD4+ T cells against the percentage of CD45RO+ − of CD4+ T cells for Cohort B (Pearson *r* = 0.33, *p* < 0.0001). (**C**) Scatter plot of the percentage of FAS+ CD4+ T cells against the percentage the percentage of CD31+ CD4+ T cells (Pearson *r* = 0.29, *p* < 0.001). Data are displayed as the percentage of CD4+ T cells, and correlations were determined using Pearson product–moment correlation coefficients for PD patients and caregivers combined *(n* = 136) from Cohort B. Best-fit lines were determined by linear regression. (JPEG 36 kb)

High resolution (TIFF 325 kb)

Fig. S3 CD4+ T cell and Teff phenotypes are associated with UPDRS-III score. **a** Scatter plot of the percentage of CD45RO+ CD4+ T cells against UPDRS-III score (Pearson *r* = 0.35, *p* = 0.003, *n* = 69). **b** Scatter plot of the percentage of FAS+ CD4+ T cells against UPDRS-III score (Pearson *r* = 0.24, *p* = 0.043, *n* = 70). **c** Scatter plot of the percentage of CD31+ CD4 T cells against UPDRS-III (Pearson *r* = −0.49, *p* < 0.001, *n* = 70). **d** Scatter plot of the percentage of integrin α4β7+ CD4+ T cells against UPDRS-III (Pearson *r* = −0.29, *p* = 0.02, *n* = 58). **e** Scatter plot of the percentage of CD31+ Teff against UPDRS-III score (Pearson *r* = −0.47, *p* < 0.0001, *n* = 69). **f** Scatter plot of the percentage of α4β7+ Teff against UPDRS-III score (Pearson *r* = −0.32, *p* = 0.017, *n* = 57). Data are displayed as the percentage of CD4+ T cells (**a-d)** or CD25+CD127+CD4+ Teff (**e** and **f**) and correlations were determined using Pearson product–moment correlation coefficients. Best-fit lines were determined by linear regression. (JPEG 54 kb)

High resolution (TIFF 29131 kb)

ESM 4 (DOCX 21 kb)
