# Supplementary figures and images for: CD4+ Regulatory and Effector/Memory T Cell Subsets Profile Motor Dysfunction in Parkinson’s Disease
Source: J Neuroimmune Pharmacol. 2012 Oct 11;7(4):927–38. doi: 10.1007/s11481-012-9402-z (PMC3515774; doi:10.1007/s11481-012-9402-z)

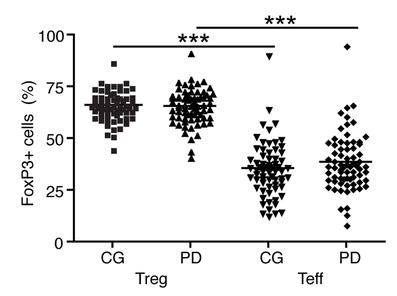

Supplement: Supplementary file 1 — The percentage of FoxP3+ Treg and Teff from PD patients and caregivers. Data are the percentages of FoxP3 positive Treg and Teff with medians (horizontal lines). Significant differences among groups were determined by Kruskal-Wallis nonparametric ANOVA, and pair-wise comparisons determined by Dunn’s multiple comparison’s post-hoc analysis where ***p ≤ 0.001. (JPEG 14 kb) [file 11481_2012_9402_Fig5_HTML.jpg]

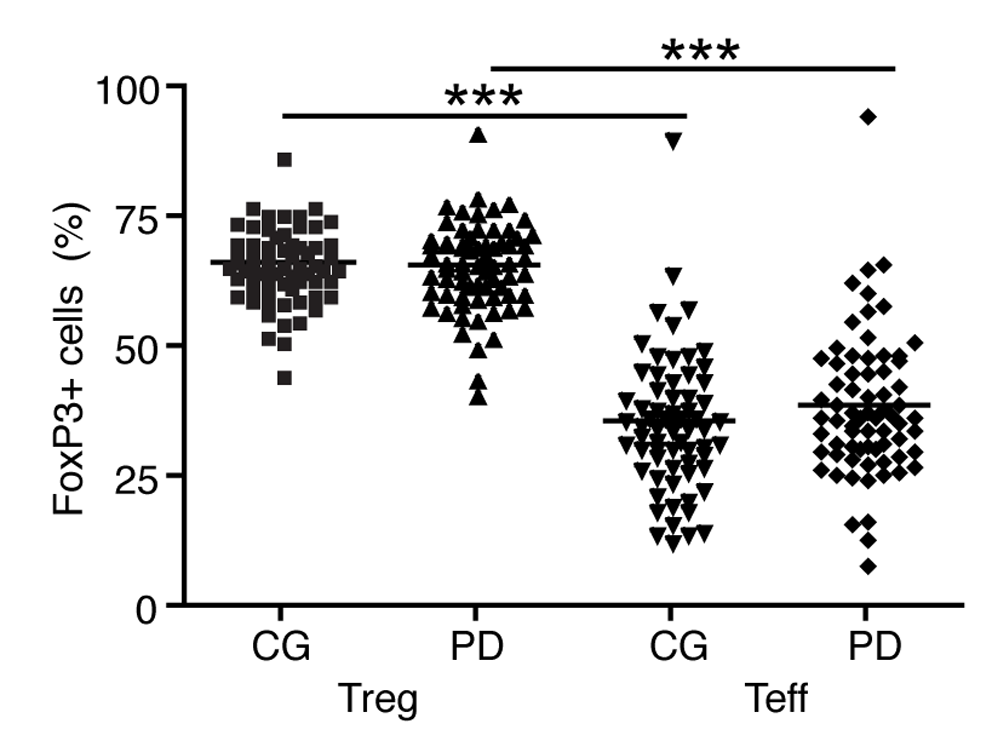

Supplement: Supplementary file 2 — High resolution (TIFF 151 kb) [file 11481_2012_9402_MOESM1_ESM.tif]

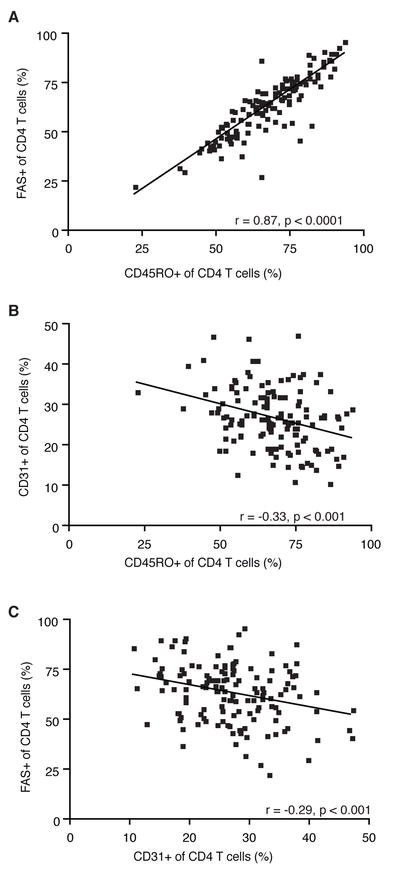

Supplement: Supplementary file 3 — The percentages of CD45RO+, FAS+ and CD31+ CD4+ T cells are correlative. a Scatter plot of the percentage of FAS+ CD4+ T cells against the percentage of CD45RO+ of CD4+ T cells for Cohort B (Pearson r = 0.87, p < 0.001). b Scatter plot of the percentage of CD31+ CD4+ T cells against the percentage of CD45RO+ − of CD4+ T cells for Cohort B (Pearson r = 0.33, p < 0.0001). (C) Scatter plot of the percentage of FAS+ CD4+ T cells against the percentage the percentage of CD31+ CD4+ T cells (Pearson r = 0.29, p < 0.001). Data are displayed as the percentage of CD4+ T cells, and correlations were determined using Pearson product–moment correlation coefficients for PD patients and caregivers combined (n = 136) from Cohort B. Best-fit lines were determined by linear regression. (JPEG 36 kb) [file 11481_2012_9402_Fig6_HTML.jpg]

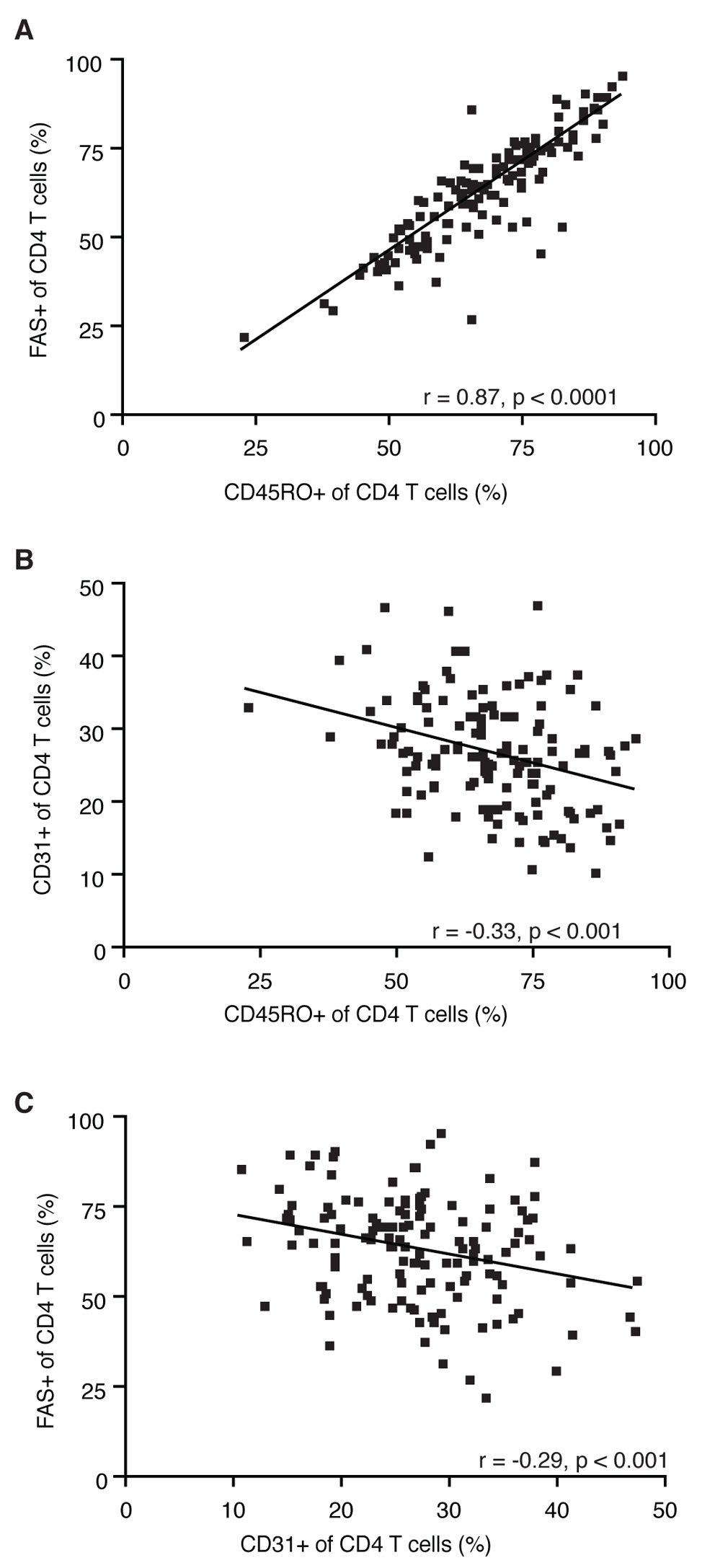

Supplement: Supplementary file 4 — High resolution (TIFF 325 kb) [file 11481_2012_9402_MOESM2_ESM.tif]

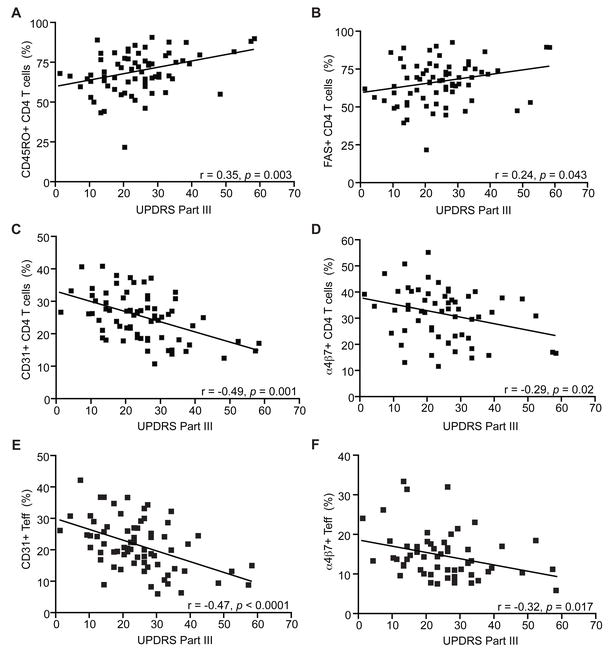

Supplement: Supplementary file 5 — CD4+ T cell and Teff phenotypes are associated with UPDRS-III score. a Scatter plot of the percentage of CD45RO+ CD4+ T cells against UPDRS-III score (Pearson r = 0.35, p = 0.003, n = 69). b Scatter plot of the percentage of FAS+ CD4+ T cells against UPDRS-III score (Pearson r = 0.24, p = 0.043, n = 70). c Scatter plot of the percentage of CD31+ CD4 T cells against UPDRS-III (Pearson r = −0.49, p < 0.001, n = 70). d Scatter plot of the percentage of integrin α4β7+ CD4+ T cells against UPDRS-III (Pearson r = −0.29, p = 0.02, n = 58). e Scatter plot of the percentage of CD31+ Teff against UPDRS-III score (Pearson r = −0.47, p < 0.0001, n = 69). f Scatter plot of the percentage of α4β7+ Teff against UPDRS-III score (Pearson r = −0.32, p = 0.017, n = 57). Data are displayed as the percentage of CD4+ T cells (a-d) or CD25+CD127+CD4+ Teff (e and f) and correlations were determined using Pearson product–moment correlation coefficients. Best-fit lines were determined by linear regression. (JPEG 54 kb) [file 11481_2012_9402_Fig7_HTML.jpg]

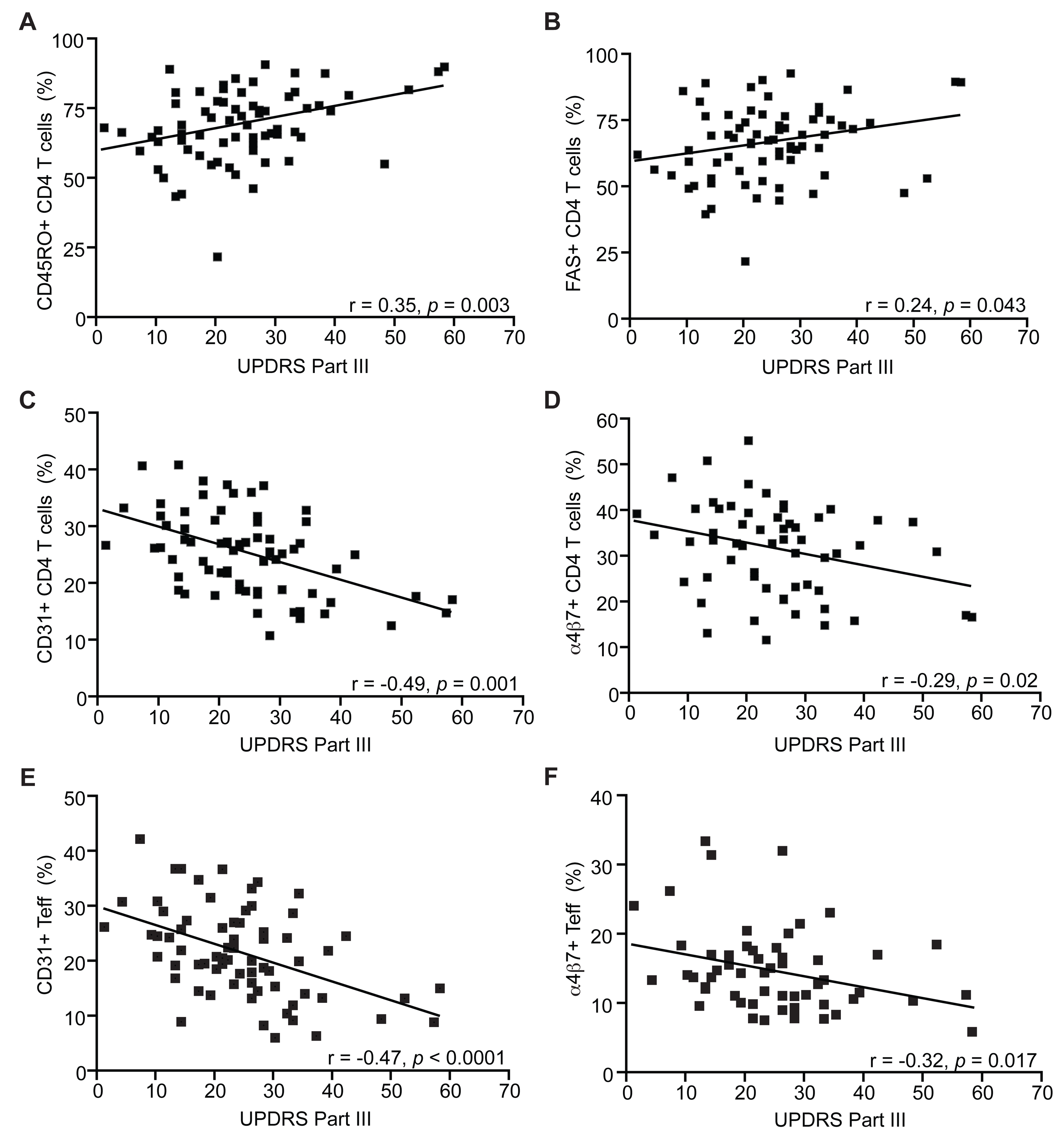

Supplement: Supplementary file 6 — High resolution (TIFF 29131 kb) [file 11481_2012_9402_MOESM3_ESM.tif]
