# Supplementary material for: CD4+ Regulatory and Effector/Memory T Cell Subsets Profile Motor Dysfunction in Parkinson’s Disease
Source: J Neuroimmune Pharmacol. 2012 Oct 11;7(4):927–38. doi: 10.1007/s11481-012-9402-z (PMC3515774; doi:10.1007/s11481-012-9402-z)
Supplement: Supplementary file 7 — (DOCX 21 kb) [file 11481_2012_9402_MOESM4_ESM.docx]

**Table S1. Descriptive data of Cohorts A and B**

|  | Cohort A | | | | | Cohort B | | | | |
| --- | --- | --- | --- | --- | --- | --- | --- | --- | --- | --- |
|  | Caregivers | | PD patients | |  | Caregivers | | PD patients | |  |
|  | n | Mean ± SD | n | Mean ± SD | *p* value | n | Mean ± SD | n | Mean ± SD | *p* value |
| Age (yrs.) | 28 | 61 ± 10 | 38 | 64 ± 8 | 0.14 | 57 | 64 ± 11 | 72 | 67 ± 10 | 0.12 |
| Disease duration (yrs.) | - | - | 37 | 4.5 ± 2.5 | NA | - | - | 72 | 4 ± 2.5 | NA |
| H&Y stage | - | - | 34 | 2 ± 0.5 | NA | - | - | 71 | 2 ± 1 | NA |
| UPDRS-III score | - | - | 36 | 26 ± 10 | NA | - | - | 70 | 24 ± 11 | NA |
|  | Caregivers | | PD patients | |  | Caregivers | | PD patients | |  |
|  | n | Percent | n | Percent | *p* value | n | Percent | n | Percent | *p* value |
| Gender, male | 30 | 27 | 41 | 78 | < 0.0001 | 65 | 23 | 72 | 75 | <0.0001 |
| Race, Caucasian | 30 | 100 | 41 | 90 | NA | 65 | 98 | 72 | 99 | NA |
| Job with pesticides | 30 | 13 | 41 | 25 | > 0.05 | 65 | 8 | 71 | 25 | 0.006 |
| Exposure to pesticides | 30 | 47 | 40 | 78 | 0.04 | 59 | 39 | 70 | 70 | 0.05 |
| Job with chemical solvents | 30 | 10 | 41 | 37 | 0.024 | 63 | 10 | 71 | 27 | 0.002 |
| Job with heavy metals | 30 | 7 | 41 | 17 | > 0.05 | 63 | 3 | 71 | 13 | 0.04 |
| Job with other chemical fumes | 29 | 4 | 40 | 13 | > 0.05 | 63 | 14 | 71 | 31 | 0.01 |

**Table S2. Phenotypic Analysis of CD4+ T cells and subsets from PD patients compared to caregivers in Cohort A.**

|  |  | Caregivers | | | | PD patients | | | | *p* values | |
| --- | --- | --- | --- | --- | --- | --- | --- | --- | --- | --- | --- |
| Population | Population Subset | N | Mean ± SEM (%) | Median (%) | Range (%) | N | Mean ± SEM (%) | Median (%) | Range (%) | Mann-Whitney | Benjamini-Hochberg |
| CD4+ T cells | CD45RO+ | 23 | 65.0 ± 2.31 | 66.2 | 41.0-80.2 | 29 | 74.7 ± 1.8 | 75.3 | 48.8-91.0 | 0.002 | 0.008 |
|  | CD45RA+ | 23 | 25.5 ± 2.1 | 24.4 | 9.8-47.6 | 29 | 16.9 ± 1.6 | 16.3 | 4.8-36.8 | 0.002 | 0.008 |
|  | FAS+ | 23 | 58.4 ± 2.8 | 52.8 | 37.9-79.7 | 30 | 69.6 ± 2.3 | 70.0 | 39.1-88.4 | 0.005 | 0.012 |
|  | CD31+ | 23 | 28.2 ± 1.9 | 26.6 | 11.1-43.0 | 30 | 22.9 ± 1.8 | 20.8 | 8.9-42.7 | 0.025 | 0.035 |
|  | Integrin β7+ | 23 | 36.6 ± 2.7 | 36.4 | 17.7-60.2 | 30 | 29.8 ± 1.4 | 30.0 | 15.9-5.0 | 0.025 | 0.035 |
|  | CD25+ | 27 | 9.1 ± 0.3 | 8.8 | 6.1-13.5 | 33 | 9.7 ± 0.4 | 9.6 | 6.9-16.5 | 0.28 | 0.33 |
|  | CD127+ | 27 | 59.1 ± 1.88 | 60.4 | 36.8-75.8 | 33 | 61.5 ± 1.5 | 64.0 | 42.7-76.3 | 0.36 | 0.36 |
| CD4+CD25+ CD127+ Teff | CD45RO+ | 23 | 87.1 ± 1.3 | 89.8 | 72.3-96.6 | 28 | 90.4 ± 1.2 | 91.8 | 70.1-97.1 | 0.03 | 0.07 |
|  | CD45RA+ | 23 | 4.0 ± 0.6 | 3.1 | 0.7-10.3 | 28 | 2.9 ± 0.8 | 1.8 | 0.5-17.7 | 0.02 | 0.07 |
|  | FAS+ | 24 | 90.6 ± 2.1 | 94.4 | 48.9-97.6 | 31 | 94.1 ± 1.1 | 95.9 | 68.8-99.6 | 0.08 | 0.10 |
|  | CD31+ | 25 | 24.0 ± 1.5 | 23.3 | 9.2-36.4 | 30 | 20.3 ± 1.8 | 18.6 | 7.0-38.9 | 0.12 | 0.12 |
|  | Integrin β7+ | 25 | 21.4 ± 1.6 | 20.5 | 6.3-39.8 | 31 | 18.3 ± 1.7 | 16.6 | 7.0-59.7 | 0.06 | 0.08 |
| CD4+CD25+ CD127- Treg | CD45RO+ | 23 | 71.5 ± 1.1 | 82.6 | 71.5-92.6 | 28 | 86.4 ± 1.0 | 87.8 | 73.9-93.3 | 0.012 | 0.07 |
|  | CD45RO- | 23 | 9.5 ± 1.0 | 9.2 | 1.8-21.4 | 28 | 7.1 ± 0.9 | 5.8 | 1.5-20.4 | 0.04 | 0.07 |
|  | FAS+ | 24 | 51.4 ± 83.5 | 94.3 | 82.5-2.0 | 31 | 71.3 ± 88.5 | 96.6 | 86.9-1.3 | 0.04 | 0.07 |
|  | CD31+ | 25 | 8.7 ± 22.4 | 41.4 | 24.9-1.8 | 30 | 6.6 ± 18.3 | 41.3 | 20.8-1.9 | 0.10 | 0.11 |
|  | Integrin β7+ | 25 | 10 ± 14.2 | 25.5 | 15.6-0.9 | 31 | 8.3 ± 12.3 | 21.9 | 13.0-0.6 | 0.03 | 0.07 |
